# Factors that influence the plant use knowledge in the middle mountains of Nepal

**DOI:** 10.1371/journal.pone.0246390

**Published:** 2021-02-11

**Authors:** Durga Kutal, Ripu M. Kunwar, Kedar Baral, Prabhat Sapkota, Hari P. Sharma, Bhagawat Rimal

**Affiliations:** 1 University of Wisconsin-Whitewater, Whitewater, WI, United States of America; 2 Ethnobotanical Society of Nepal, Kathmandu, Nepal; 3 Massey University, Palmerston North, New Zealand; 4 Department of Forest and Soil Conservation, Kathmandu, Nepal; 5 Central Department of Zoology, Institute of Science and Technology, Tribhuvan University, Kathmandu, Nepal; 6 College of Applied Sciences (CAS)-Nepal, Tribhuvan University, Kathmandu, Nepal; Ilia State University, GEORGIA

## Abstract

An account of total of 58 plant species including 57 genera and 43 families was reported as useful in ethnomedicine from semi-structured questionnaire survey to the 76 participants of Kaski and Baitadi districts, Nepal. Fieldwork and participatory meetings were carried out between September 2017 and January 2018. A total of 419 emic use reports including 150 from Kaski and 269 from Baitadi were reported from 58 ethnomedicinal plant species. Each species was reported for 2–43 use reports and each participant recorded 1–12 use reports. About 25% (n = 104) use reports were associated with the treatment of digestive system disorders followed by 83 for general complaints. Of the species assessed, 53 species had IASc value < 0.25 and only five species had > 0.25. Species *Swertia chirayita*, *Paris polyphylla*, *Bergenia ciliata*, *Valeriana jatamansi* and *Centella asiatica* with > 0.25 IASc were found to be highly consented; however they were incongruent between the sample groups and sites. Divergent plant use knowledge specific to each sample district and group was corresponding to the heterogeneity of socio-economy and culture of the sites. Gender, ethnicity, household economy and food availability of the respondents were leading factors affecting the plant use knowledge. Despite the sites were relatively homogenous in eco-physiography, they possessed the distinct plant use knowledge, hinted that the socio-economic factors are more explanatory in plant use knowledge.

## 1. Introduction

Nepal has the largest elevational gradient in the world [1] extending from tropical alluvial plains as low as 59 meters above sea level (m asl) in the lowland Tarai to the Words’ highest summit Mt. Everest (8,848 m asl) [2]. Vertically, there are five distinct eco-physiographical regions: the Himalaya (23% of total area, and above 5,000 m asl), High Mountains (20%, between 3,000 and 5,000 m asl), the Middle Mountains (30%, between 1,000 and 3,000 m asl), Siwalik Hills (12.8%, between 500 and 1,000 m asl) and the flat, lowlands of Tarai (13.7%, < 500 m asl) [3]. The country has only 14.75% arable land [4] that lies mostly at lowlands and hills. Less availability of arable land accelerates the human pressure on remaining agricultural land [5] and surrounding open-access forests [6].

When there is little arable land and it is acerbated by geo-ecological constraints [7], indigenous livelihood strategies include outmigration, off-farm activities, animal husbandry, summer grazing, and collection, use, and trade of medicinal plants [8, 9]. Thus, livelihood strategies at rural areas to be precise in mountains and hills of Nepal are greatly influenced by different ethnoecological environments defined by the availability of resources [10], geography of the region [11], economy and socio-culture [12, 13], and weather [14]. It is hypothesized that the apparent species and accessible sites are more frequently foraged. Philip and Gentry [15] stated that the availability of a plant is linked to its relative importance to a given community. Higher use value of plant relates to its higher abundance and thus is more likely to be collected than the rarely encountered ones [16]. The largest, most dominant, and most available plants have the highest use values, not because they are intrinsically useful, but simply because they are salient [15, 17]. However, the use value of salient plant is weakly associated with socio-economic factors [18].

Subsets of socio-economic factors such as settlement, population, family size, gender, age, ethnicity, education, economy, and occupation have effect on knowledge of plant use [19–21]. Studies have demonstrated that ethnobotanical knowledge increases with an individual’s age [22] and length of residence [23]. Women are more familiar with the medicinal values of local flora [20]. Native and indigenous people possess rich ethnobotanical knowledge [24]. Thus, the cultural variables seem more essential in explaining knowledge of plant collection and use [25] in addition to the sustainability of plant resources. However, change in lifestyle as a result of globalization, increasing population, land-use change, and climate warming worsen the rural livelihood [25, 26]. A continuous outmigration foments a decline in the number of healers and indigenous knowledge holders [27, 28], resulting in weakened indigenous knowledge and use systems [29]. Documentation and conservation of indigenous knowledge is therefore a prerequisite in order to conserve the biodiversity, promote rural livelihood and help build the community resilience against climate change [30].

In this study, we evaluated the cross-cultural plant use knowledge of two different ethnic groups (Gurung dominated, and Brahmin-Chhetri and others (BCO) dominated) how their socio-economic (gender, age, household size, education, economy, land possesses, livestock ownership, and food availability), and cultural (ethnicity, length of residence and length of healing practice) variables influence plant use. A total of 11 socio-economic and 2 physiographic independent variables (distance from home to forest and home to health center) were used for assessing the knowledge of medicinal plant use (number of species used and ailments treated) as dependent variable. We hypothesized that the collection and use of medicinal plant is not random, it is associated with socio-economy and culture of the area. We also assumed that the use value of plants is less influenced by plants’ ecological salience feature. For intercultural study, we compared the knowledge of plant collection and use of two heterogeneous communities inhabiting homogenous eco-physiographical setting. We hypothesized that there is a difference in average medicinal species used between two sample groups.

## 2. Materials and methods

### 2.1 Study area

**Fig 1:**
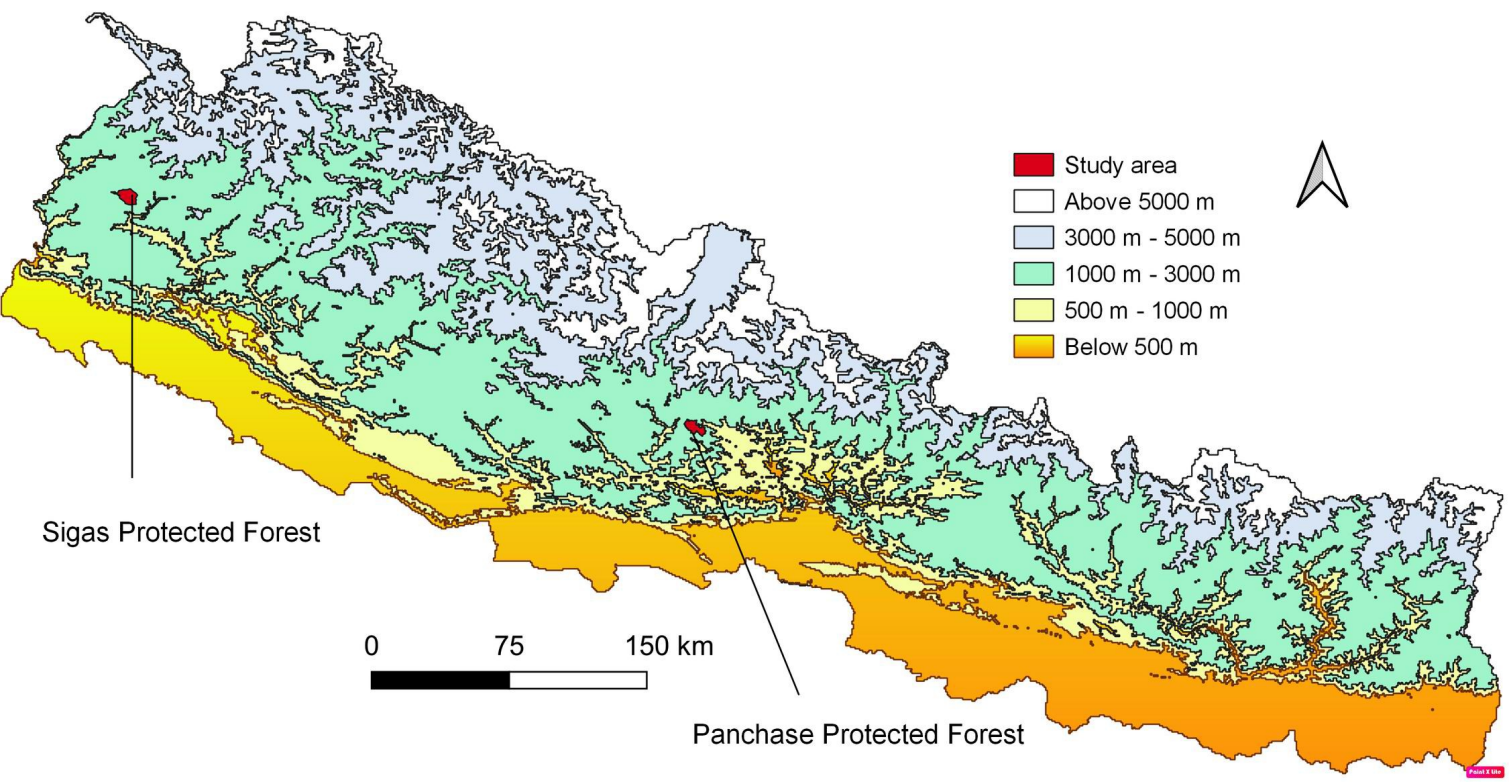
The map shows the intensive study area with elevation and physiographic regions. Data for this map was downloaded from ICIMOD [31].

This study was carried out in Sigas area (29° 26.13’– 29° 30.65’ N; 80° 41.68’– 80° 47.26’ E) of Baitadi and Panchase area (28° 12’– 28° 18’ N; 83° 45’– 83° 57’ E) of Kaski district, Nepal (Fig 1). Both sites (Sigas and Panchase) have similar eco-physiography since they represent protected forests of the country, are a part of bigger conservation landscape, mid-hill physiography with moderate road access and have protected forest as a resource base for local livelihood. The mountain peaks of both areas (Sigas-Dhura and Panchase temple) are regarded as sacred and religious and annually thousands of pilgrims access the sites. Data for study area map was downloaded from ICIMOD [31] and QGIS 3.10.12 was used to prepare the map.

However, Sigas, a part of the Kailash Sacred Landscape is recently declared protected forest and Panchase, a part of Chitwan-Annapurna Landscape was declared as a protected forest in 2012. The protected forest is one of the forest types, designated by the ministry of forest and soil conservation, government of Nepal for conservation of biodiversity and promotion of local livelihood. The Panchase protected forest, situated at the junction of three districts Kaski, Syangja and Parbat with an elevation range of 900–2517 m represents mid-hill physiography [31], boasts of a total of 613 flowering plant species including 107 non-timber forest products [32, 33] and 113 orchid species [34]. It represents an important ecological zone that is less addressed amongst the country’s protected area system and is only one biodiversity corridor linkage of lowland (Chitwan–Nawalparasi) and Annapurna Himalaya range (Chitwan–Annapurna Conservation Landscape). The area has great biological, cultural and religious diversities as well as natural beauty [35, 36]. The dominant caste of this area is Gurung.

The Sigas protected forest is a recently declared protected forest of Baitadi district, ranged within 1400–2850 m and is characterized by Oak-Laurel forest and rich socio-cultural heritage. This area is established to conserve the biodiversity and culture of Sigas-Dhura, Baitadi district [37]. The area is dominated by Brahmin-Chhetri castes. Baitadi district itself is renowned for socio-culturally designated sacred peaks [38]. The legacy of sacredness has been valued to designate the site as protected forests and conservation landscape and to have a conservation effect on culture, heritage and ecology. Sigas, Baitadi district represents the lower and southern part of the Kailash Sacred Landscape (KSL). The KSL is a sacred trans-boundary and tri-national landscape of Nepal, India and China. Agriculture, medicinal plant collection and trade and traditional healing are the major occupations in Sigas, Baitadi whereas in Panchase, Kaski agriculture, business, foreign employment, summer grazing, medicinal plant collection and trade and traditional healing are common [21, 39–41] however, the agriculture contributes the most in both sites. Foreign employment and summer grazing are secondary livelihood strategies at Panchase, Kaski whereas in Sigas, Baitadi collection of forest products including medicinal plants is next to agriculture.

### 2.2 Survey and data collection

Division Forest offices Kaski and Baitadi granted written permissions to survey the forest and local communities living around the forest. We obtained Florida Atlantic University IRB approval and district forest office level approval for carrying out interview respectively in Baitadi and Kaski district." Prior informed oral consent was obtained from the interview participants. The sample communities of both districts were composed of sedentary farmers. Traditional medicinal practice is their subordinate occupation. A total of 76 (50 men and 26 women) traditional medicinal practitioners, 38 in each district were interviewed based on snowball sampling and local references, village secretaries and divisional forest office staffs. Total 38 traditional medicinal practitioners in Sigas were from Shivalinga, Sigas, Gajari, Chaujham, Thalakanda, Dhungad and Shikharpur villages, Baitadi and the same number in Panchase from Arthur Dandakharka, Bhadaure Tamagi, Chapakot, Chitre and Ramja Deurali villages of Kaski district. Inventory-based semi-structured questionnaire (S1 File) interviews were carried out with the help of local assistants between September 2017 and January 2018.

In the interview, the participants were asked to list the three most important medicinal plants and their use details for traditional healing. Information regarding demography (age, gender, household size) and socio-economy (occupation, education, livestock, land ownership, length of residence, distance of home from district center, nearest health post, and forest) were sough while interviewing (Table 1). Interviews were supplemented with other investigative techniques, such as participant observation, *walk-in-the-woods*, and informal meetings [42]. Informal meetings were held during the evenings while staying with local communities, and sometimes with tea vendors. While participating in the *walk-in-the-woods*, voucher specimens of the species that could not be identified in the field were collected by participants and field assistants and processed and deposited at the Plant Laboratory and Herbarium (KATH), Lalitpur, Nepal, for future reference. Earlier studies carried out in and around study area [9, 13, 32, 40, 41, 43, 44] were used as a taxonomic reference of plant species. Plant taxon was verified by using Plants of the World Online http://www.plantsoftheworldonline.org.

**Table 1:** Variables and their type used in this study.

Explanatory variables	Variable type
Age (Year)	Continuous
Family member (Number)	Continuous
Land possess (Ropani)	Continuous
Livestock (Number)	Continuous
Long been settled (Year)	Continuous
Traditional medicinal practice (Year)	Continuous
Distance home-forest (Km)	Continuous
Distance home-health center (Km)	Continuous
Ethnicity	Categorical: Gurung (37), Brahmin, Chhetri and Others (39)
Gender	Categorical: Male (50), Female (26)
Education	Categorical: Illiterate (42), Literate (34)
Food availability	Categorical: < 6 months (42), > 6 months (34)
Economy	Categorical: Poor (16), Medium (44), Rich (16)
Site	Categorical: Baitadi (38); Kaski (38)

### 2.3 Data analysis

Matching information (use reports) from at least three respondents was considered a common response for quantitative analysis [19]. To determine the influence of socio-economic factors, we used the value of useful species, which represents the sum of all useful species an informant knew. The local perception, classification and uses of plants (Emic use) [45] were later grouped into etic uses for convenient analyses following International Classification of Primary Care (ICPC) [46]. ICPC is a suitable standard for cross-cultural comparisons [47]. To identify the proportion of culturally important species in each study district, the Index of Agreement on Species (IAS) was calculated following [48]:

(ns−nu)/(ns−l),

whereby, ns is the number of use reports of a given species mentioned by all the participants, and nu is the number of use types attributed to that species. IAS was corrected to Index of Agreement on Species consensus (IASc) for the number of participants who knew a use for the species through the formula:

IASc=IAS×(Pu/Pt)

where, Pu represents the number of participants who reported a use, and Pt equals the total number of participants interviewed about the species. IASc values vary between 0 and 1, with 0 representing no agreement and 1 total agreement. In this paper, we determined the proportion of plant species with an IASc value > 0.25; this value was chosen as an arbitrary cutoff point for culturally important species following Vandebroek [49]. The result of IASc was supported by informant consensus factor (FiC). Naturally, both assess the consensus; however, the first assesses the consensus at species level whereas the latter evaluates at use types/category level. FiC was calculated as:

nur−nspp.used/nur−1

where, nur shows the number of use reports while nspp shows the number of species used [48].

Ecologically salient species are available, easy to access, frequent and abundant [50]. The salience index is very important in ethnobotanical studies because of its usefulness in identifying species of better quality for specific purposes [18, 51]. We used salience index featured with the ecological characteristics (availability (rare = 0, moderate = 1, common = 2), access (limited = 1, easy = 2), abundance (hardly found = 0, rare = 1, common = 2) and life form (annual herb = 1, perennial herb = 2, shrub = 3, tree = 4) of each species in order to evaluate the relationship of plant use values against the salience features [52].

### 2.4 Statistical analysis

We grouped the socio-cultural and demographic data into nominal/categorical variable: (1) gender, (2) ethnicity, (3) education, (4) food availability, (5) site, and (6) household economy and continuous variable: (1) age, (2) household size, (3) livestock size, (4) land size, (5) length of residence, (6) years of healing practice, (7) distance from home to forest, and (8) distance from home to health center. Cross-cultural analysis was made by (1) gender: male and female, (2) ethnicity: Gurung and Brahmin-Chhetri and others (BCO), (3) education: literate and non-literate, (4) food availability: < 6 and > 6 months, (5) site: Kaski and Baitadi, and (6) household economy: rich, medium and poor. Multiple linear regression models were used to explore how socio-cultural variables interact among themselves and with the knowledge of plant collection, use, and management intensity. Two-way ANOVA was used to further know how these important categorical variables in combination affect the dependent variable (number of plant used and number of ailments treated). We compared the population averages from the independent samples of unequal variances of the medicinal species used in Kaski and Baitadi district. Level of significance was applied at *p* ≤ 0.1. All the analyses were performed in R studio in R 3.4.1 (R Development Core Team 2017).

## 3. Results and discussion

### 3.1 Medicinal plants and uses

A total of 58 medicinal plant species including 57 genera and 43 families were reported as highly useful in study area. Asteraceae, Urticaceae, Apiaceae, Lamiaceae were the dominant plant families contributing 4, 4, 3, and 3 species respectively. Of 58 useful plant species, 36 were from in Kaski and 49 from Baitadi, with common 28 species. Bhattarai et al. [32] had reported 45 medicinal plants from 44 genera, being useful for treatment of 34 ailments (use types). Whereas a large number of medicinal plants 102 were reported as useful in herbal remedy in Baitadi district after consulting 57 traditional healers around Sigas area [13]. From 58 ethnomedicinal plant species, our 76 participants reported a total of 419 emic use reports. There were 150 use reports from Kaski (3.94 person^**-1**^ use reports) and 269 from Baitadi (7.07 person^**-1**^ use reports). The use reports were grouped into 55 use types and 14 use categories for convenient analysis. Each species was reported for 2–43 use reports and each respondent recorded 1–12 use reports.

### 3.2 Informant consensus on use types

About 25% (n = 104) use reports were associated with the treatment of digestive system disorders (indigestion 66, gastritis 21, dysentery 4, diarrhea 3, stomachache 3, constipation 3, gall stone 4) followed by 83 use reports for general complaints (fever 54, toothache 10, dehydration 8, typhoid 5, inflammation 4, and headache 2) and 55 use reports for respiratory ailments (cough and cold 48, sinusitis 3, bronchitis 2 and pneumonia 2). The use medicinal plants for abdominal complaints was found to be predominant in Gurung dominated communities in Kaski district [53]. The frequent use of medicinal plants to cure digestive disorders could be attributed to the high preponderance of complaints associated with the digestive system. This account aligns with the government report stating the prevalence of diarrhea and dysentery in rural villages. The health condition was acerbated by food deficiencies in Baitadi [51] and by contaminated water in Kaski [54].

This finding was supported by informant consensus factor (FiC). Of the 55 use types, we sorted out use types with use reports ≥ 10 and evaluated their FiC (Table 2). The highest FiC (0.83) was reported for fever followed by 0.80 for indigestion, stomachache and gastritis and 0.77 for antidoting and bites. This gives us idea that the general ailments are treated by available medicinal plants and this could be a result of cultural learning. Out of 76 traditional medicinal practitioners, 59 (77%) learnt from families and communities. Apprenticeship learning is valued in traditional medicine for treating general ailments [55]. Apprenticeship under the tutelage of senior practitioners is common in both districts [13, 56]. General knowledge about popular medicinal uses of plants is commonly shared in apprenticeship through cultural learning.

**Table 2:** Use categories, types and reports and informant consensus factor (FiC).

Use category	Use type	Use reports	Useful species	Species	FiC
General	Fever	54	10	*B*. *aristata*, *T*. *chebula*, *O*. *tenuiflorum*, *S*. *wallichii*, *P*. *frutescens*, *R*. *ellipticus*, *C*. *verutum*, *C*. *asiatica*, *P*. *polyphylla*, *S*. *chirayita*	0.83
	Toothache	10	5	*C*. *dactylon*, *J*. *regia*, *M*. *esculenta*, *S*. *aromaticum*, *Z armatum*,	0.55
Digestive	Indigestion, gastritis, stomachache	97	20	*A*. *calamus*, *A*. *aspera*, *A marmelos*, *A*. *racemosus*, *A*. *vera*, *B*. *ciliata*, *C*. *asiatica*, *C*. *plantaginea*, *C*. *rufa*, *C*. *verutum*, *D*. *boryana*, *J*. *regia*, *M*. *spicata*, *P*. *emblica*, *P*. *polyphylla*, *P*. *roxburghii*, *U*. *dioica*, *V*. *jatamansi*, *W*. *fruticosa*, *Z*. *armatum*	0.80
Blood, Spleen, lymph	Antidoting Snakebite	19	5	*A*. *calamus*, *C*. *asiatica*, *C*. *dactylon*, *C*. *plantaginea*, *P*. *polyphylla*	0.77
	Cuts & Wounds	10	5	*A*. *adenophora*, *C*. *montana*, *P*. *polyphylla*, *P*. *roxburghii*, *S wallichii*,	0.55
Circulatory	Jaundice	14	5	*C*. *dactylon*, *C*. *reflexa*, *Q*. *lanata*, *S*. *aromaticum*, *Z mauritiana*	0.69
Respiratory	Cough & Cold	48	16	*A*. *calamus*, *B*. *aristata*, *B*. *ciliata*, *C*. *verutum*, *C*. *angustifolia*, *C*. *tamala*, *E*. *foetidum*, *E*. *officinalis*, *O*. *tenuiflorum*, *P*. *frutescens*, *Q*. *lanata*, *R*. *ellipticus*, *R*. *wallichiana*, *T*. *chebula*, *V*. *jatamansi*, *Z*. *armatum*	0.68
Skin	Skin disease	27	14	*A*. *cachemerica*, *A*. *indica*, *A*. *vera*, *C*. *asiatica*, *C*. *reflexa*, *D*. *albicalyx*, *G*. *diversofolia*, *G*. *hirta*, *J*. *regia*, *P*. *emblica*, *P*. *polyphylla*, *S*. *nigrum*, *U*. *dioica*, *V*. *jatamansi*,	0.5
Musculo-skeleton	Fracture and sprain	19	11	*A*. *evecta*, *B*. *ciliata*, *C*. *angustifolia*, *C*. *reflexa*, *D*. *albicalyx*, *D*. *longofolia*, *G*. *diversifolia*, *O*. *sativa*, *R*. *nepalensis*, *T*. *contorta*, *U dioica*,	0.44

### 3.3 Agreement on species consensus

Of the species assessed, 53 species had IASc value < 0.25 and only five species had > 0.25 (S2 Table). Species *Swertia chirayita* (Roxb.) H.Karst., *Paris polyphylla* Sm., *Bergenia ciliata* (Haw.) Sternb., *Valeriana jatamansi* Jones ex. Roxb. and *Centella asiatica* (L.) Urb. were found to be highly consented. We considered IASc > 0.25 as a cutoff value for identifying the highly consented species. Medicinal plants and their elevation range (*B*. *ciliata*: 900–1700 m, *C*. *asiatica*: 500–2100 m, *P*. *polyphylla*: 1500–3500 m, *S*. *chirayita*: 1500–2500 m and *V*. *jatamansi*: 1400–3600 m) and distribution throughout Nepal from east to west [57–59] hinted that these species are more accessible than others leading to frequent collection in mid-hills. Accessible plant species are often considered more useful than less abundant or less accessible [11, 17]. This posited and admitted that plants are collected non-randomly and the ones of proximity and familiarity are frequently collected.

**Fig 2:**
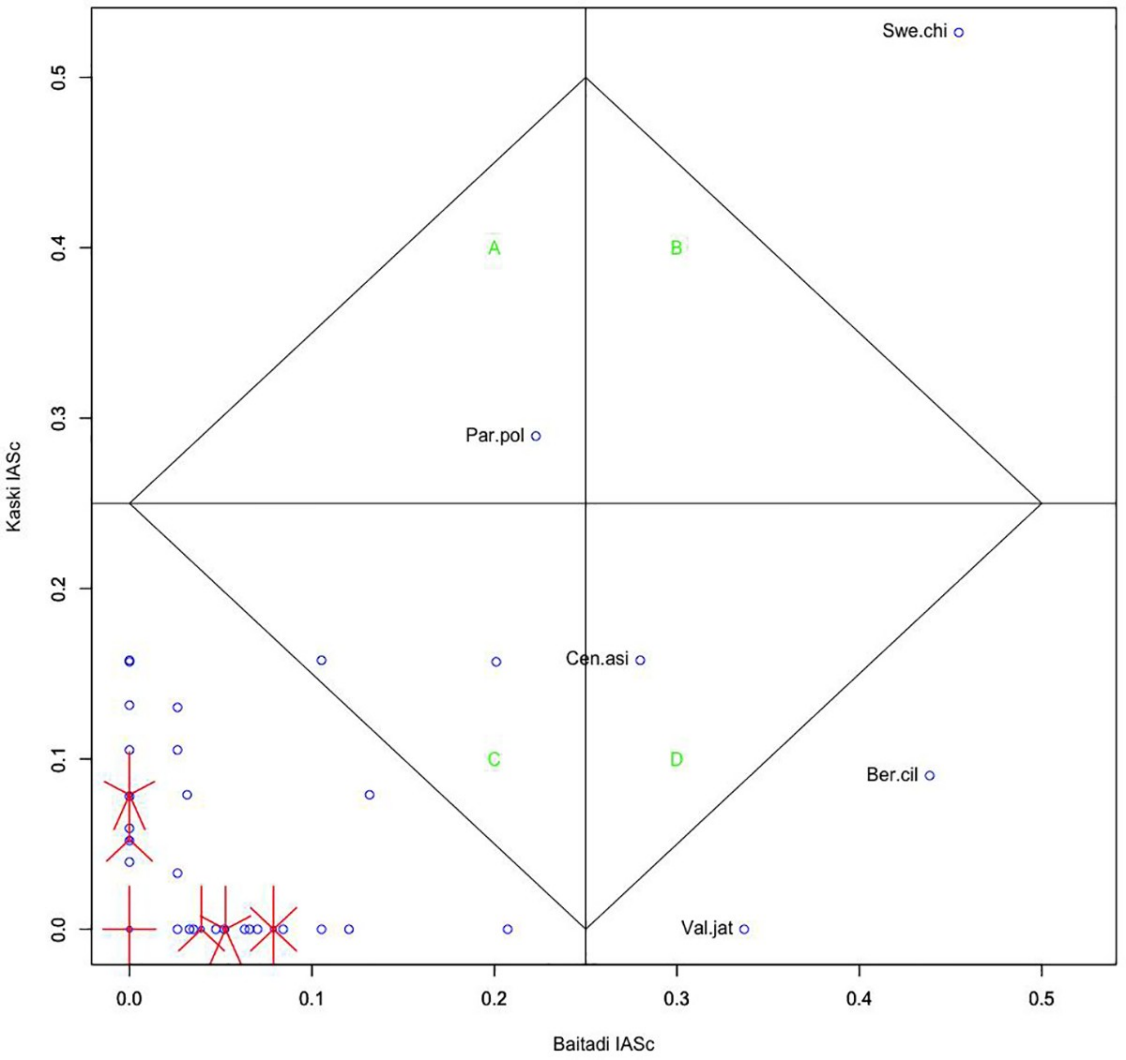
Cultural consensus matrix of two groups (participants from Panchase, Kaski and Sigas, Baitadi).

Of the species, *B*. *ciliata*, *V*. *jatamansi* and *C*. *asiatica* were frequently used (> 0.25 IASc) only in Baitadi (Quadrant D) whereas species *P*. *polyphylla* with > 0.25 IASc was common in medicinal folklore in Kaski (Quadrant A). The comparison of informant agreement values (IASc) between the Baitadi and Darchula districts was different but insignificant (*p*  =   0.413). Despite being in the similar ecological landscape, the medicinal plants were differently valued, perhaps because of different ethnic groups, accessibility, use values, and livelihood strategies. This result was supported by the fact that only one species *S*. *chirayita* appeared in Quadrant B was commonly used in both districts. Quadrant B has common species between the districts with the highest IASc values whereas the Quadrant C the common species with the lowest IASc values. Contrarily, the medicinal plant species with the highest IASc values and common only in Kaski are occupied in Quadrant A and that species common only Baitadi are located in Quadrant D. The species positioned in Quadrant C were insignificant in uses and consensus (Fig 2).

Most of the species with low IASc value indicated that there were very few species with higher species consensus, offering the prospects of diversification hypothesis [60]. Local people introduce second priority and easily available species in their ethnomedicinal repertoire since the several options are available. It is also plausible that human communities that inhabit ecosystems diverse in species, diversify their repertoire and use different species [61], resulting in diverse knowledge of plant use. This is possible in this case since both Kaski and Baitadi, mid-hill districts of Nepal, are rich in biodiversity and serve variety of plants for folklore. The mid-hill region (also known as Middle Mountain) is characterized by sub-tropical and temperate bioclimates with a great variety of terrain types, ecosystems and wildlife [62], flowering plants [63, 64] and medicinal plants [32]. Because of the variety of medicinal plants available, people have tried using 20 medicinal plants only for the treatment of digestive system disorder.

However, *P*. *polyphylla*, *B*. *ciliata and V*. *jatamansi* were highly consented and competent in treatment of indigestion. Over 50 percent population lives in hills and mountains and it is complicated by persistent outmigration to cities [65]. Outmigration is rife in Panchase, Kaski [35, 54]. Farmlands are abandoned while emigration. Warming is a biggest threat in the hills and mountains [66]. A recent study shows the mid and far western hills and mountains are more sensitive to and affected by warming [67]. Conservation of medicinal plants and their associated traditional knowledge is in jeopardy in trajectory of poverty, migration, land use change and climate change [68, 69]. The hills and mountains not only have the highest percentage of poverty (42.3%) on average, but it is also increasing [70].

### 3.4 Plant use knowledge and socio-economic variables

Only one species *S*. *chirayita* was highly consented in both district indicated that the plant use knowledge was specific to the district groups and culturally divergent. The divergent and idiosyncratic knowledge revealed that the sample groups are unique knowledge holder. The plant use knowledge was specific to each district, corresponding to the heterogeneity of socio-economy and culture of the sites. The difference (*p* = 0.0003–0.95) in plant use knowledge was governed by socio-economy and culture (Table 3).

**Table 3:** Correlation coefficients of the variables used.

SN	Explanatory variables	Statistical values	Number of ailments treated (*p*)	Number of species used (*p*)
1	Age (Year)	Min 36, Max 90, Mean 65, St Dev 9.87	0.836	0.692
2	Family member (Number)	Min 3, Max 21, Mean 7.6, St Dev 2.98	0.934	0.093*
3	Land possess (Ropani)	Min 2, Max 50, Mean 16.7, St Dev 9.87	0.178	0.142
4	Livestock (Number)	Min 0, Max 21, Mean 5.2, St Dev 4.01	0.631	0.592
5	Long been settled (Year)	Min 18, Max 200, Mean 125, St Dev 44.24	0.641	0.918
6	Traditional medicinal practice (Year)	Min 0, Max 80, Mean 21.5, St Dev 17.93	0.956	0.500
7	Distance home-forest (Km)	Min 0.25, Max 3, Mean 0.91, St Dev 0.65	0.945	0.788
8	Distance home-health center (Km)	Min 0.25, Max 3, Mean 1.12, St Dev 0.78	0.0003***	0.278
9	Ethnicity	Gurung (37)	0.713	0.040*
		BCO (39)		
10	Gender	Male (50)	0.091*	0.760
		Female (26)		
11	Education	Literate (34)	0.711	0.888
		Illiterate (42)		
12	Food availability	< 6 months (42)	0.360	0.056*
		> 6 months (34)		
13	Economy	Poor (16)	0.062*	0.183
		Medium (44)		
		Rich (16)		
14	Site	Baitadi	0.105	0.007**
		Darchula		
15	Economy x Gender		0.590	0.761
16	Ethnicity x Food availability		0.129	0.374

* Low significant

** Moderate significant

*** High significant, Confidence level 90%.

Of the variables tested, distance from home to health center (continuous variable) was the most significant (*p* = < 0.0003) followed by categorical variables (ethnicity: *p* = 0.040, food availability: *p* = 0.056, household economy: *p* = 0.062, gender: *p* = 0.091 and family member: *p* = 0.093). People use locally available medicinal plants for their primary health care because of the limited access and health care facilities available in the village. Summer grazing, herding and collection of medicinal plants and their uses is still persistent with some adaptation [71] among Gurung ethnic group of Kaski, Panchase area [69]. Gurung were mountain dwellers, herder of sheep, swidden farmers and trans-himalayan traders [72]. While herding, summer grazing, and collecting the medicinal plants, local people share the knowledge of plant identification, uses, and management. These traditional practices help hold and share the higher knowledge among the respondents of Kaski and revealed the significant difference (*p* = 0.040) in knowledge of medicinal plant collection and use. The average number of medicinal plants used was 5.1±2.25 whereas the number of medicinal plants used among Gurung communities was higher (5.8±2.4) and among Brahmin, Chhetri and others (BCO) was less (4.48±1.9). This could be a reason of frequent access to the forest. The average distance between home and forest for Gurung communities was 0.8±0.48 km while that for BCO was 1.01±0.78. Typically forest is adjacent to the Gurung village [73] that is a veritable natural pharmacopoeia. It has the purported potential to cure almost any afflictions. We did not find the significant difference in the number of livestock possesses, however the Gurung communities held higher number of livestock 6.43±4.05 than BCO (4.07±3.67).

The medicinal plant collection was also influenced by food availability (*p* = 0.056) and household economy (*p* = 0.062). Over 55% households have food insufficiency for > 6 months. There is little arable land and it is curtailed by the hills and steep terrain. Because of the insufficient food, household heads often go to nearby forests for collection of plants to complement the household food, economy and healthcare. Nearby forests, considered as open free access commodity [6] are often foraged for collecting fuelwood, vegetable, food plants, and medicinal plant collection and grazing.

Furthermore, utilization of medicinal plants between male and female participants was different (*p* = 0.09), yet it was not statistically significant. This could be a reason of the gendered division of labor in Nepal where males are often engaged in summer grazing, livestock herding, and extraction of plants from remote and rural areas whereas women are more likely to be involved in managing local resources that are available nearby. Pfeiffer and Butz [74] reported that plant use is differentiated with men and women due to resource access and their social roles. Education level, possession of livestock and land, length of residence and age of the respondents were not significantly associated with the ethnomedicinal knowledge. A weak association of these factors and plant use knowledge could be attributed to the (non-random) selective samples and the limited sharing of information. The secrecy of ethnomedicinal knowledge is a common practice [16] and traditional healers hardly share their knowledge to outsiders with the belief that effectiveness would decrease if knowledge is revealed [75]. Traditional healers generally believe that the medicines would lose their efficacy if too many people know about their use. Age also did not reveal a significant difference in the response, even though the 80–89 years group was more knowledgeable [22]. As education is associated with the loss of ethnobotanical knowledge in indigenous communities [76], the relation of education and ethnomedicinal knowledge in our study area was nonexistent. Combination of categorical variables also did not affect the number of medicinal plants used and the number of ailments treated. The number of medicinal plant species used between Kaski and Baitadi districts was found statistically significantly different (*p* = 0.007). The difference led that the knowledge was specific to each district, corresponding to the heterogeneity of socio-economy and culture of the sites. This result rejected our null hypothesis and supported that the plant use knowledge is associated with local socio-economy and culture.

### 3.5 Plant salient features and use values

**Fig 3:**
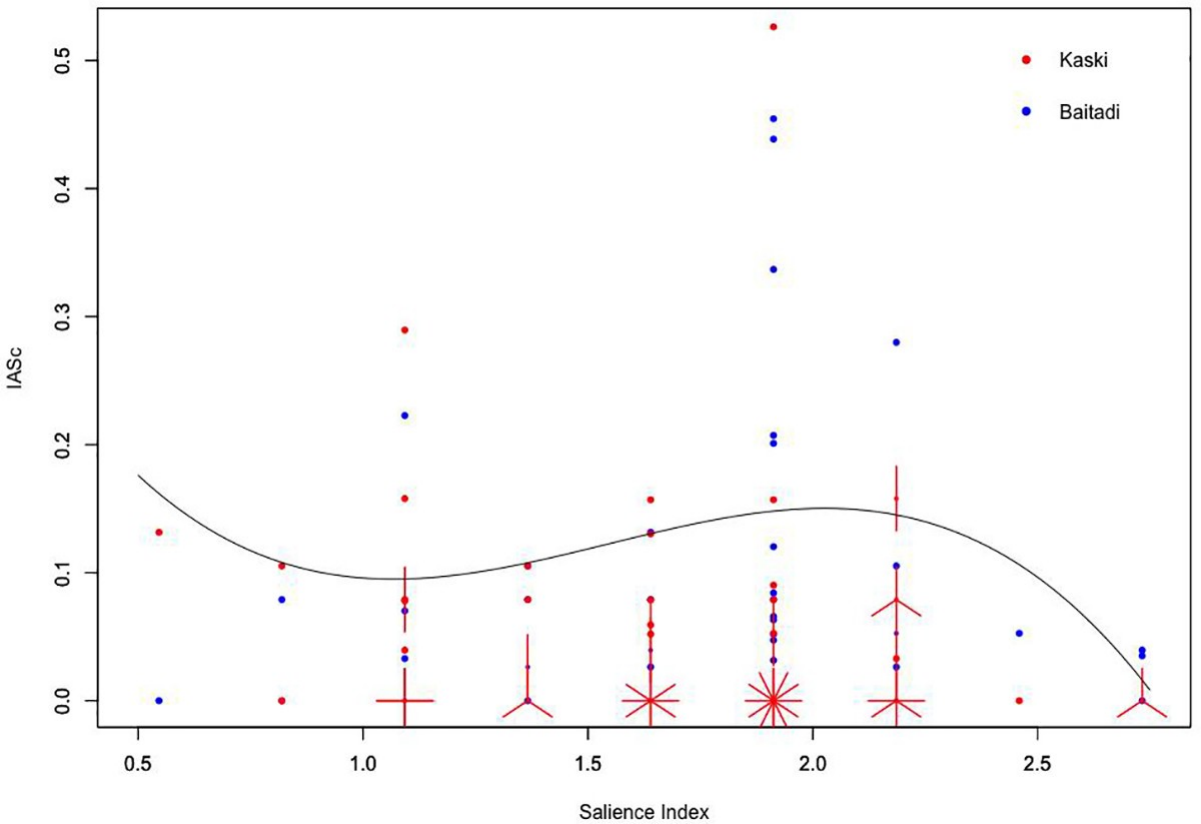
Regression of plant use knowledge consensus (IASc) of participants along the gradients of plant salience index.

*Pinus roxburghii* Sarg., *Rhododendron arboreum* Sm., *Taxus contorta* Griff., *Ficus palmata* Forssk., etc. were useful tree species of the area but they possessed the insignificant IASc value. However, they were the species with the highest salience index. There was weak relationship with plant use values and plant salient features (Fig 3), hinted that plant ecological traits are not adequate in explaining the factors of plant selection and use. Weak association of plant use values and salience index may be outweighed by the direct harvesting and/or by the popularity of the particular uses of that species. Species popularity and usefulness are related [18], meaning that the knowledge of plant use is less influenced by availability of plants (salient feature) and more dependent on cultural preoccupancy and resource quality. *P*. *polyphylla* (*Satuwa*) is one of the top five useful species in the area, and is popular for its medicinal uses since people believe that its seven leaves are useful for curing seven ailments (cultural preoccupancy) [13]. Moreover, *B*. *ciliata* rhizomes for indigestion and *S*. *chirayita* leaves for fever are folkloric [77].

The healers in study area were picky to have the right species, composition and procedures when preparing ethnomedicine. Such directed harvesting influenced the collection of medicinal plants regardless of their distribution and salient features. Sousa et al. [18] found the weak association of salient plants and socio-economic factors. According to Thomas et al. [11] the phyto-sociological indices (abundance, availability) were more associated for non-medicinal use category (wood, fuel and construction uses).

## 4. Conclusions

Although the few accessible medicinal plant species possessed the higher use values, the overall medicinal plants’ use values were convergent to the socio-cultural variables. Gender, ethnicity, food availability, family size and household economy significantly affected the number of plants used and ailments treated. Moreover, direct harvesting and or the popularity of the particular uses of that species explained the higher plant use values. The knowledge of medicinal plant species use was associated with socio-economy and culture and significantly different at district level. To sum up, the knowledge of plant use in mid-hills of Nepal seems to follow a pattern according to available useful plants as well as the socio-cultural tradition. However, the latter prevails. Strategies acknowledging socio-economy, culture, and local ecosystem would be appropriate for conservation of medicinal plants and traditional knowledge in the mid-hills of Nepal.

## Supporting information

S1 File Questionnaire survey form. (DOCX)

S1 Table (CSV)

S2 Table IASc value and salience index of plants. (CSV)
