# Supplementary material for: Factors that influence the plant use knowledge in the middle mountains of Nepal
Source: PLoS One. 2021 Feb 11;16(2):e0246390. doi: 10.1371/journal.pone.0246390 (PMC7877619; doi:10.1371/journal.pone.0246390)
Supplement: S1 File — (DOCX) [file pone.0246390.s001.docx]

cg';Gwfg ;xefuLtf ;Demf}tf

s}nfz klaq e' kl/lw If]qsf] dfgj / ag:ktL ;DaG3L k/Dk/fut 1fgsf] ;j]{If0f, cWoog, laZn]Zf0f / ;+/If0f

l/k' s'j/

km\nf]l/8f P6nflG6s o'lge/l;l6

e' la1fg laefu, ##$#!, af]sf /|of6g, km\nf]l/8f

kmf]gM ! $&) %%$ %^$^ Od]nM rkunwar@fau.edu

lh lk cf] aS; !(@@%, sf7df8f}, g]kfn

kmf]gM ! (*$( !#) ^)@

Od]nM ripukunwar@gmail.com

o; cWoogsf] p2]Zo s}nfz klaq e' kl/lw If]qsf] dfgj / ag:ktL ;DaG3sf] k/Dk/fut 1fgsf] ;j]{If0f, cWoog, laZn]Zf0f / ;+/If0f ug{' xf] . o;f] ul//xbf logsf] jftfj/l0fo, hnjfo', kof{j/l0fo, ;fdflhs, cfyL{s, ;f:s[lts, ef}uf]lns, / e'pkof]u ;DalGw cGt/;DjG3sf] cWoog / laZn]Zf0f Pa+ dfgj, ag:ktL / k|s[tLsf] lbuf] ;DaGwsf] cg'zGwfg Pa+ AofVof ug{nfuL o; If]qdf a;f]af; ug]{ AolQmx? hf] ag:ktL tyf hl8a'6Lsf] af/]df k|z:t 1fg /fVg' x'G5, pxfx?;u+ e]6 ul/g]5 / dfgj, ag:ktL / k/Dk/fut 1fg / ltgsf] cGt/;DaGwsf] uxg cWoog cg'zGwfg ul/g]5 .

o; cWoog s'g} klg k|of]uzfnf, pTkfbg, tyf a]r lavgsf] pb]Zon] k|eflat 5}g . of] cWoog :yfgLo, /fli6|o / cGt/fli6|o dfGotf cg'?k kl/rfnLt 5 . of] laz'2 k|fl1s / cg'zGwgfTds x'g]5 . o;sf nfuL d]/f] pb]Zo ufpsf a}B, a[b a[bfx? e]6\g], plgx?;+u ePsf] dfgj, ag:ktL / hl8a'6L af/]sf] :yfklt Pa+ ;DalGwt k/Dk/fut 1fgsf] ;j]{If0f / cWoog ul/ To;sf] tYof+s sf]; tof/ ug]{ / n]v /rgfx? k|sflzt u/]/ AolQ,m ;d'bfo, ;dfh, / /fHoNfO{ ;';'lrt ub}{ u/fpb} cGt/fli6|o :t/df g]kfnsf] pkl:ytL b]vfpg' klg xf] .

cfh d tkfO{;+u tkfO{sf] lhjgsf] dxTjk'0f{ la?jfx? s] x'g, sxf kfO{G5, s] s] gfdn] lrlgG5, nufPt cGo ;Dk'0f{ hfgsf/L lng] k|oTg ug]{]5' . olb tkfO{ /fhL x'g'x'G5 eg] d of] s'/fsfgL Pa+ cGt{jftf{ cufl8 a9fpg rfxG5' / ;Demgfsf] nfuL l6kf]6x? sfkLdf n]Vg] cg'dtL rfxG5' . ;'rgf uf]Kotfsf] nfuL gfdnfO{ sf]8df ablng] 5 / o; cWoog, k|sfzg kZrft oL ;Dk'0f{ l6kf]6 gf]6x? gi6 ul/g]5 . tkfO{sf] O{R5f ljgf tkfO{sf] gfd ;fj{hlgs ul/g] 5}g .

o; cGt{jftf{df efu lnb} ubf{ xfdL b'a} efu lng] cyjf lbg] kIFnfO{ s'g} cfly{s nfe xfgL x'g] 5}g . oL dfyLsf s'/fx?df ;dy{g hgfpg] / o; cGt{jftf{df efu lng] jf glng] tkfO{sf] :j larf/ xf] . of] cGt{jftf{ sl/a cfwL 306fsf] x'g]5 . o; k|ZgfjnLdf ;f]lwPsf k|Zgx? tkfO{nfO{ c;xh nfu]df lar}df kgL tkfO{ cGt{jftf{ 5f]8\g ;Sg' x'G5 .

tkfO{nfO{ o; cWoog ;DjlGw s]lx yk hfgsf/L rflxPdf dnfO{ ;f]Wg ;Sg' x'g]5 cyjf dfyLsf] d]/f] 7]ufgfdf ;Dks{ /fVg ;Sg' x'g]5 .

tkfO{ o; cGt{jftf{df ;xefuL eP/ dnfO{ ;xof]u ug{ dGh'/ x'g'x'G5 <

gd:sf/ k|ZgfjnL ldtL

sf]8 g+ of] 7fpsf] prfO e' cIff+z, b]zfGt/

uf lj ; 7]ufgf

!. AolQmut ljj/0f

gfd k9fO{ ln+u pd]/

d'Vo k]zf wd{ af]Ng] efiff M ! @ #

tkfO{ oxf a:g' ePsf] stL eof] < tkfO{ a;fO{ ;/]/ cfpg' ePsf] eP sxf af6 cfpg' ePsf] xf] -lhNnf, 7fp<

3/kl/jf/ ;+Vof ;a} 3/df x'g'x'G5 < ljb]z

tkfO{sf] 3/df vfgf / cGg sf] pknAwtf ^ dlxgf eGbf sd, ^ b]lv !@ dlxgf ;Dd, !@ dlxgf eGbf al9

aif{el/sf] 3/sf] vr{sf] Aoa:yf -! b]vL ^ ;Dd_ s[ifL -a}b]l;s_ /f]huf/ Hofnf dhb'/L Aofkf/ ahf/ hl8a'6L cGo

3/sf] cfDbfgL ÷cGg vfgf df hl8a'6L ;+sngsf] lx:;f -k|ltztdf_

tkfO{sf] hUuf hldg 3/ ufO{ j:t' cGo

3/ b]lv h+un;Ddsf] b'l/ -;do_ 3/ b]lv ;b/d'sfd;Ddsf] b'l/ -;do_ 3/ b]lv :jf:Yo s]Gb|;Ddsf] b'l/ -;do_

@. a}B ;Djlw pkrf/ / hl8a'6Lsf] k|of]u

tkfO{ a}B ;Djlw pkrf/ / hl8a'6Lsf] k|of]u ub}{ cfpg' ePsf] 5 <

slt ;do eof] a}B ;Djlw pkrf/ ug{ nfUg' ePsf]] < sf] sxfaf6 s;/L l;Sg' eof] <

tkfO{sf] a'af, xh'/a'af a}B ;DjlGw sfd ug'{ x'GYof] < tkfO{sf] 5f]/f, 5f]/L jf sf]xL ub}{ x'g'x'G5 ls

#. hl8a'6L ;+sng

tkfO{ hl8a'6L hGo la?jfx? sxf sxfaf6 ;+sng ug{' x'G5< -l6s nufpg]_ h+un v]tjf/L ahf/

k|fo sf] hfG5 hl8a'6L hGo la?jfx? ;+sng ug{ hl8a'6L ;+sng d"Vo ÷k|fylds sfd xf] <

$. la/fdL / /f]u

tkfO{sxf k|fo s:tf dflg;x? w]/}h;f] pkrf/sf] nfuL cfp5g< -d'Vo ! df l6s nufpg]_

ul/a dWood wlg k'?if dlxnf cGo

hghftL afx'g If]qL blnt afn aRrf o'jf o'jtL a'9f a'9L

:yflgo lhNnfsf 6f9f 6f9f af6 klxnf] k6s c:ktfnsf] pkrf/ kl5 bfxf]/ofP/

tkfO{sxf cfpg] la/fdLsf] rfk a9|bf] p:t} 36|bf]

d'Vo s] s:tf /f]usf] lgbfgsf] nfuL tkfO{sxf+ cfp5g< ! @ #

tkfO{n] clxn];Dd pkrf/ u/fP/ cTolws ;kmn x'g'ePsf] /f]usf] gfd ! @ #

tkfO{n] pkrf/ u/fpbf k|of]u ug]{ cGo d'Vo lrhx? -hl8a'6L la?jf afx]s_ ! @ #

Uffpsf] k|d'v :jf:Yo ;d:of s] xf] < ! @ #

tkfO{sf] uf lj ; df tkfO{h:t} pkrf/ u/fpg] c? slt hgf 5g / sf] sf], ltgsf] 7]ufgf………

!

@

#

tkfO{sxf kl5Nnf] k6s cfpg'ePsf] la/fdLsf] hfgsf/L – slxn], sxfaf6, sf], s] sfdsf] nfuL

^. tkfO{sf] lhjgsf] % ;a}eGbf dxTjk'0f{ lj?jfx? sf] cWoog -s[kof qmdz ! dfyL af6 tn;Dd el/;s] kl5 @, #, $, % eg'{ xf]nf_

| **tkfO{sf] lhjgsf] % ;a}eGbf dxTjk'0f{ lj?jfx?^-#_^** | **:**jf:Yo/cf}iflwo, ;fdflhs/;f:s[lts, cfly{s/lhljsf]kfh{g, kof{j/l0fo -k\|ofKt eP/_, ef}uf]lns lx;fan] -c? gkfpg] eP/_ | | | | |
| --- | --- | --- | --- | --- | --- |
|  | !. | @. | #. | $. | %. |
| s] sfdnfO{ dxTjk'0f{^-#_^ |  |  |  |  |  |
| of\] slxn] s;/L / s] sfddf k\|of]u ug]{ |  |  |  |  |  |
| of] la?jfsf] k\|of]u dfyLsf dWo] s'g ! sf/0f xf]nf |  |  |  |  |  |
| of] la?jf slxn]af6 k\|of]udf cfPsf] xf]nf, a'af xh'/a'afn] k\|of]u ug'{eof] |  |  |  |  |  |
| of] la?jf slxn] sxf af6 ;+sng ug]{ |  |  |  |  |  |
| h+undf kfpg] eP s'g  **;/sf/L,;Fd'bfoLs,AolQut** |  |  |  |  |  |
| of] ;a}eGbf glhs kfpg] 7fFp, b'l/ |  |  |  |  |  |
| of] la?jf gePsf] eP csf]{ s'g la?jf dxQjk'0f x'GYof] of] sfdsfnfuL^-@_^ |  |  |  |  |  |
| of] klg gePsf] eP csf]{ s'g lj?jf^-!_^ |  |  |  |  |  |
| dfyLsf] ! g+ sf] la?jfsf] cGo effifdf o;sf] gfd |  |  |  |  |  |
| elaiodf o;sf] pknA3tf  **s'g} Ps** -k\|z:t, l7s}, Go'g_ |  |  |  |  |  |
| dfyLsf] ! g+ sf] la?jfsf **o;sf] bf]z\|f] k\|of]u^-@_^** |  |  |  |  |  |
| dfyLsf] ! g+ sf] la?jfsf **o;sf] cGo k\|of]u^-!_^** |  |  |  |  |  |
| o;sf] s]lx ;fdfhLs syg, ;f:s[lts lsDabGtL, k\|rng …  -pbfx/0fsf] nfuL ljjfx, Gjf/g, dbf{ kbf{, kf:gL, cflb_, k\|of]udf  s'g} af/, dlxgf, ;do, 7fp hfthftL, cGo s]lx laz]if 5 ? |  |  |  |  |  |
| dfyL elgPsf dWo] s'g} ug{, vfg, k\|of]u ubf{ g/fd\|f] x'g] s]lx? |  |  |  |  |  |
| o;sf]] lbuf] ;+/If0fnfO{ s]ug'{{ knf{ | ! | ! | ! | ! | ! |
| cGo cf}iflw hGo lj?jfx? s] s] 5g / ltgsf] k\|of]u | ! |  | $ |  | |
|  | @ |  | % |  | |
|  | # |  | ^ |  | |
