# Supplementary material for: Factors that influence the plant use knowledge in the middle mountains of Nepal
Source: PLoS One. 2021 Feb 11;16(2):e0246390. doi: 10.1371/journal.pone.0246390 (PMC7877619; doi:10.1371/journal.pone.0246390)
Supplement: Attachment — Submitted filename: Response to reviewers.12.18.docx [file pone.0246390.s004.docx]

Response to reviewers/editors comments

1. We have revised the acknowledgements and removed any funding related text.

1.1 We want to keep the funding statement as follows.

Funding Statement

Ripu Kunwar received an Early Career Grant (EC-212R-18) from National Geographic Society (USA) for field travel in Baitadi. Kedar Baral and Prabhat Sapkota received grants from divisional forest offices for field visits. But the grants did not have any additional role in the study design, data collection and analysis, decision to publish, or preparation of the manuscript. The specific roles of the authors are given in the ‘author contributions’.

2.

We have added “Division Forest offices Kaski and Baitadi granted written permissions to survey the forest and local communities living around the forest. We obtained Florida Atlantic University IRB approval and district forest office level approval for carrying out interview respectively in Baitadi and Kaski district.".

was added at the beginning of the method section.

3. a

Data for study area map was downloaded from ICIMOD [31] and QGIS 3.10.12 was used to prepare the map.

b. Fig 1. Caption was restructured.
