# Supplementary material for: Factors that influence the plant use knowledge in the middle mountains of Nepal
Source: PLoS One. 2021 Feb 11;16(2):e0246390. doi: 10.1371/journal.pone.0246390 (PMC7877619; doi:10.1371/journal.pone.0246390)
Supplement: Peer review history

# Peer review: Decision Letter 0

**DOI:** 10.1371/journal.pone.0246390.r001

**Authors:** Rainer W. Bussmann

18 Nov 2020

PONE-D-20-31088

Factors that influence the plant use knowledge in the middle mountains of Nepal

PLOS ONE

Dear Dr. Kunwar,

Thank you for submitting your manuscript to PLOS ONE. After careful consideration, we feel that it has merit but does not fully meet PLOS ONE’s publication criteria as it currently stands. Therefore, we invite you to submit a revised version of the manuscript that addresses the points raised during the review process.

Please submit your revised manuscript by Jan 02 2021 11:59PM. If you will need more time than this to complete your revisions, please reply to this message or contact the journal office at plosone@plos.org. When you're ready to submit your revision, log on to https://www.editorialmanager.com/pone/ and select the 'Submissions Needing Revision' folder to locate your manuscript file.

Please include the following items when submitting your revised manuscript:

- A rebuttal letter that responds to each point raised by the academic editor and reviewer(s). You should upload this letter as a separate file labeled 'Response to Reviewers'.
- A marked-up copy of your manuscript that highlights changes made to the original version. You should upload this as a separate file labeled 'Revised Manuscript with Track Changes'.
- An unmarked version of your revised paper without tracked changes. You should upload this as a separate file labeled 'Manuscript'.

If you would like to make changes to your financial disclosure, please include your updated statement in your cover letter. Guidelines for resubmitting your figure files are available below the reviewer comments at the end of this letter.

If applicable, we recommend that you deposit your laboratory protocols in protocols.io to enhance the reproducibility of your results. Protocols.io assigns your protocol its own identifier (DOI) so that it can be cited independently in the future. For instructions see: http://journals.plos.org/plosone/s/submission-guidelines#loc-laboratory-protocols

We look forward to receiving your revised manuscript.

Kind regards,

Rainer W. Bussmann

Academic Editor

PLOS ONE

Journal Requirements:

When submitting your revision, we need you to address these additional requirements.

1. Please ensure that your manuscript meets PLOS ONE's style requirements, including those for file naming. The PLOS ONE style templates can be found at

https://journals.plos.org/plosone/s/file?id=wjVg/PLOSOne_formatting_sample_main_body.pdf and

https://journals.plos.org/plosone/s/file?id=ba62/PLOSOne_formatting_sample_title_authors_affiliations.pdf

2.  Thank you for stating the following in the Acknowledgments Section of your manuscript:

"The author RMK is thankful to Early Career Grant of National Geographic Society, US

384 for providing partial supports for field work in Baitadi. Hari Sharma, Suresh Subedi,

385 Prem Bhat, Shivraj Bhatt, Devendra Kunwar, Sony Baral, Ram Acharya are

386 acknowledged for their supports for fieldwork and data analysis. Thanks are also due to

387 Laxmi Mahat and Christopher Leboa for their feedback on earlier versions."

i) We note that you have provided funding information that is not currently declared in your Funding Statement. However, funding information should not appear in the Acknowledgments section or other areas of your manuscript. We will only publish funding information present in the Funding Statement section of the online submission form.

ii) Please remove any funding-related text from the manuscript and let us know how you would like to update your Funding Statement. Currently, your Funding Statement reads as follows:

 "The funders had no role in study design, data collection and analysis, decision to publish, or preparation of the manuscript.".

 * Please include your amended statements within your cover letter; we will change the online submission form on your behalf.

3. We note that [Figure 1] in your submission contain map images which may be copyrighted. All PLOS content is published under the Creative Commons Attribution License (CC BY 4.0), which means that the manuscript, images, and Supporting Information files will be freely available online, and any third party is permitted to access, download, copy, distribute, and use these materials in any way, even commercially, with proper attribution. For these reasons, we cannot publish previously copyrighted maps or satellite images created using proprietary data, such as Google software (Google Maps, Street View, and Earth). For more information, see our copyright guidelines: http://journals.plos.org/plosone/s/licenses-and-copyright.

We require you to either (1) present written permission from the copyright holder to publish these figures specifically under the CC BY 4.0 license, or (2) remove the figures from your submission:

1.     You may seek permission from the original copyright holder of Figure [1] to publish the content specifically under the CC BY 4.0 license.  

We recommend that you contact the original copyright holder with the Content Permission Form (http://journals.plos.org/plosone/s/file?id=7c09/content-permission-form.pdf) and the following text:

“I request permission for the open-access journal PLOS ONE to publish XXX under the Creative Commons Attribution License (CCAL) CC BY 4.0 (http://creativecommons.org/licenses/by/4.0/). Please be aware that this license allows unrestricted use and distribution, even commercially, by third parties. Please reply and provide explicit written permission to publish XXX under a CC BY license and complete the attached form.”

Please upload the completed Content Permission Form or other proof of granted permissions as an "Other" file with your submission.

In the figure caption of the copyrighted figure, please include the following text: “Reprinted from [ref] under a CC BY license, with permission from [name of publisher], original copyright [original copyright year].”

2.     If you are unable to obtain permission from the original copyright holder to publish these figures under the CC BY 4.0 license or if the copyright holder’s requirements are incompatible with the CC BY 4.0 license, please either i) remove the figure or ii) supply a replacement figure that complies with the CC BY 4.0 license. Please check copyright information on all replacement figures and update the figure caption with source information. If applicable, please specify in the figure caption text when a figure is similar but not identical to the original image and is therefore for illustrative purposes only.

The following resources for replacing copyrighted map figures may be helpful:

USGS National Map Viewer (public domain): http://viewer.nationalmap.gov/viewer/

The Gateway to Astronaut Photography of Earth (public domain): http://eol.jsc.nasa.gov/sseop/clickmap/

Maps at the CIA (public domain): https://www.cia.gov/library/publications/the-world-factbook/index.html and https://www.cia.gov/library/publications/cia-maps-publications/index.html

NASA Earth Observatory (public domain): http://earthobservatory.nasa.gov/ 

Landsat: http://landsat.visibleearth.nasa.gov/

USGS EROS (Earth Resources Observatory and Science (EROS) Center) (public domain): http://eros.usgs.gov/#

Natural Earth (public domain): http://www.naturalearthdata.com/

[Note: HTML markup is below. Please do not edit.]

Reviewers' comments:

Reviewer's Responses to Questions

**Comments to the Author**

1. Is the manuscript technically sound, and do the data support the conclusions?

The manuscript must describe a technically sound piece of scientific research with data that supports the conclusions. Experiments must have been conducted rigorously, with appropriate controls, replication, and sample sizes. The conclusions must be drawn appropriately based on the data presented. 

Reviewer #1: Partly

Reviewer #2: Yes

**********

2. Has the statistical analysis been performed appropriately and rigorously? 

Reviewer #1: No

Reviewer #2: Yes

**********

3. Have the authors made all data underlying the findings in their manuscript fully available?

The PLOS Data policy requires authors to make all data underlying the findings described in their manuscript fully available without restriction, with rare exception (please refer to the Data Availability Statement in the manuscript PDF file). The data should be provided as part of the manuscript or its supporting information, or deposited to a public repository. For example, in addition to summary statistics, the data points behind means, medians and variance measures should be available. If there are restrictions on publicly sharing data—e.g. participant privacy or use of data from a third party—those must be specified.

Reviewer #1: No

Reviewer #2: Yes

**********

4. Is the manuscript presented in an intelligible fashion and written in standard English?

PLOS ONE does not copyedit accepted manuscripts, so the language in submitted articles must be clear, correct, and unambiguous. Any typographical or grammatical errors should be corrected at revision, so please note any specific errors here.

Reviewer #1: Yes

Reviewer #2: Yes

**********

5. Review Comments to the Author

Please use the space provided to explain your answers to the questions above. You may also include additional comments for the author, including concerns about dual publication, research ethics, or publication ethics. (Please upload your review as an attachment if it exceeds 20,000 characters)

Reviewer #1: The manuscript has been well developed, it is very clear and concrete. However, the central topic of the manuscript, which is the influence of socioeconomic factors on traditional knowledge, has certain deficiencies, and it is the section with the least results and discussion.

Below you will find some suggestions and comments regarding the analysis and presentation of Socioeconomic data:

I suggest that a first version of Table 2 be included in the methods to clearly explain how the socioeconomic variables analyzed were categorized, it is important to know the units in which the continuous variables have been measured, and the maximum and minimum value. This is important to understand the influence of the variables within knowledge, to analyze the variations found between both ethnic groups and to allow the future to repeat the study considering the same variables.

The authors do not present detailed information about what type of statistical analysis they have carried out with the socioeconomic information.

The current Table 2 in the results could include descriptive information of the variables, which is included in the text but it would help much more to comparatively visualize the variation in the variables within each ethnic group. A new table could allow the authors to include the results of the statistical analyzes that they carried out and that allow to see the influence of the analyzed factors, as well as the results of the comparison between both communities.

It is important to consider in the discussion the representativeness of the different levels of the factors in the analyzes, age may have little influence because possibly all the people interviewed are the same age or there is a very wide variation. It is important to consider these aspects when discussing the data.

The authors do not present any type of analysis in relation to the representativeness of the socioeconomic factors on the results of the calculated indices.

The authors present the Results and the Discussions together, and I think that this has weakened the discussion of them, mainly the results that consider the analysis of socioeconomic factors.

In the conclusions, the authors generalize the influence of the factors towards both indigenous groups, and what they should do is analyze the reason for these differences in relation to the factors analyzed. And factors are mentioned that have not been evaluated or analyzed.

In general, it would help much more if the authors included tables with the data shown in the text, in this way the comparisons are much easier to visualize.

The questionnaires used to obtain the information of the interviewees should be included as supplementary material.

Reviewer #2: To

The Editor in Chief

PLOS-one

Dear Sir/Madam

Many many thanks for sending ms for review. I am sorry for delay the ms review. Now I read manuscript thoroughly and sending comments for your kind consideration.

Title of ms: Factors that influence the plant use knowledge in the middle mountains of Nepal

Ms Number: PONE-D-20-31088

� The present study, is focused to evaluated the cross-cultural plant use knowledge of two different ethnic groups (Gurung dominated, and Brahmin-Chhetri and others (BCO) dominated) to know that how their socioeconomic (gender, age, household size, education, economy, land possesses, livestock ownership, and food availability), and cultural (ethnicity, length of residence and length of healing practice) variables influence plant use.

� In the study a total of 11 socioeconomic and 2 physiographic independent variables (distance from home to forest and home to health center) were used for assessing the knowledge of medicinal plant use as dependent variable.

� The authors have carried out study in Sigas and Panchase, having similar ecophysiography, and important part of protected forests.

I personally appreciate the efforts of the authors to collect good information in the manuscript from the unique part of the Nepal Himalayan region. I also accept the study to be considered in the journal for publication. However, I have some comments to the authors to be addressed if possible, for quality of ms and its wide readership

Comments

2.2 Survey and data collection

• In semi-structured questionnaire interviews, what was possible questions asked from the villages to be added in methodology.

• In the interview, the participants were asked to list the three most important plants for traditional healing. What was the aim behind it?

• Voucher number of each identified plant species to be given in the manuscript.

2.3 Data analysis

• Matching information (use reports) from at least three respondents was considered a common response for quantitative analysis. I hope use report is related to informants responses for plants as medicine. How it is related to quantitative analysis?.

• Emic use types and etic categories to be described,

• Ecologically salient species to be described.

3.3 Agreement on species consensus

• I am not sure how the Quadrant A, B, C, D were assessed.

3.4 Plant use knowledge and socioeconomic variables

• Limited access to health centres compels people to use local resources for their primary health care. This is appropriate to say that the resources limitation affect its use.

• The medicinal plant collection was also influenced by food availability, household economy.

• If authors suggesting insufficient food to the villagers available. What is the reason for insufficient food? and what actually they are collecting to fulfill their needs from the nearby forests to be explained for provide more information of the study area.

3.5 Plant salient features and use values

• There was weak relationship with plant use values and plant salient features. In what way the weak relationship to be explained.

Conclusion

• Conclusion section to be improved.

In my personal view these inputs may be helpful to improve manuscripts.

Thanking you

Dr Munesh Kumar

**********

6. PLOS authors have the option to publish the peer review history of their article (what does this mean?). If published, this will include your full peer review and any attached files.

If you choose “no”, your identity will remain anonymous but your review may still be made public.

**Do you want your identity to be public for this peer review?** For information about this choice, including consent withdrawal, please see our Privacy Policy.

Reviewer #1: No

Reviewer #2: No

[NOTE: If reviewer comments were submitted as an attachment file, they will be attached to this email and accessible via the submission site. Please log into your account, locate the manuscript record, and check for the action link "View Attachments". If this link does not appear, there are no attachment files.]

While revising your submission, please upload your figure files to the Preflight Analysis and Conversion Engine (PACE) digital diagnostic tool, https://pacev2.apexcovantage.com/. PACE helps ensure that figures meet PLOS requirements. To use PACE, you must first register as a user. Registration is free. Then, login and navigate to the UPLOAD tab, where you will find detailed instructions on how to use the tool. If you encounter any issues or have any questions when using PACE, please email PLOS at figures@plos.org. Please note that Supporting Information files do not need this step.

# Author comment: Author response to Decision Letter 0

**DOI:** 10.1371/journal.pone.0246390.r002

19 Dec 2020

Reviewer #1: The manuscript has been well developed, it is very clear and concrete. However, the central topic of the manuscript, which is the influence of socioeconomic factors on traditional knowledge, has certain deficiencies, and it is the section with the least results and discussion.

Below you will find some suggestions and comments regarding the analysis and presentation of Socioeconomic data:

I suggest that a first version of Table 2 be included in the methods to clearly explain how the socioeconomic variables analyzed were categorized, it is important to know the units in which the continuous variables have been measured, and the maximum and minimum value. This is important to understand the influence of the variables within knowledge, to analyze the variations found between both ethnic groups and to allow the future to repeat the study considering the same variables.

As suggested, a new table (Table 1) was added in method section with better interpretation of units of continuous variables and sub-categories of categorical variables. Maximum, minimum, mean and standard deviation (dispersion of values) values were given in Table 3. 

The authors do not present detailed information about what type of statistical analysis they have carried out with the socioeconomic information.

Multiple linear regression models and two-way anova were used. Now the MS has been improved with considering describing the models used. We are thankful to the reviewers. 

The current Table 2 in the results could include descriptive information of the variables, which is included in the text but it would help much more to comparatively visualize the variation in the variables within each ethnic group. A new table could allow the authors to include the results of the statistical analyzes that they carried out and that allow to see the influence of the analyzed factors, as well as the results of the comparison between both communities.

As suggested, a table with description of variables was used in Method section and a table with statistical results (p value, st dev, max, min and mean values) of each variables used was discussed in RD section. 

It is important to consider in the discussion the representativeness of the different levels of the factors in the analyzes, age may have little influence because possibly all the people interviewed are the same age or there is a very wide variation. It is important to consider these aspects when discussing the data. The authors do not present any type of analysis in relation to the representativeness of the socioeconomic factors on the results of the calculated indices.

We appreciated the suggestion and included the discussion of the other variables too. Earlier we focused only the discussion of statistically significant variables. 

The authors present the Results and the Discussions together, and I think that this has weakened the discussion of them, mainly the results that consider the analysis of socioeconomic factors.

We also think that because of this reason the discussion was found weak. However now we have greatly value added the discussion section. 

In the conclusions, the authors generalize the influence of the factors towards both indigenous groups, and what they should do is analyze the reason for these differences in relation to the factors analyzed. And factors are mentioned that have not been evaluated or analyzed.

Besides concluding the results for entire area, we also analyzed the results at district level and presented in conclusion, as suggested. 

In general, it would help much more if the authors included tables with the data shown in the text, in this way the comparisons are much easier to visualize.

We added one more table.

The questionnaires used to obtain the information of the interviewees should be included as supplementary material.

Questionnaire was provided as supplementary file (S1 Table).

Reviewer #2: To

The Editor in Chief

PLOS-one

Dear Sir/Madam

Many many thanks for sending ms for review. I am sorry for delay the ms review. Now I read manuscript thoroughly and sending comments for your kind consideration.

Title of ms: Factors that influence the plant use knowledge in the middle mountains of Nepal

Ms Number: PONE-D-20-31088

¬ The present study, is focused to evaluated the cross-cultural plant use knowledge of two different ethnic groups (Gurung dominated, and Brahmin-Chhetri and others (BCO) dominated) to know that how their socioeconomic (gender, age, household size, education, economy, land possesses, livestock ownership, and food availability), and cultural (ethnicity, length of residence and length of healing practice) variables influence plant use.

¬ In the study a total of 11 socioeconomic and 2 physiographic independent variables (distance from home to forest and home to health center) were used for assessing the knowledge of medicinal plant use as dependent variable.

¬ The authors have carried out study in Sigas and Panchase, having similar eco-physiography, and important part of protected forests.

I personally appreciate the efforts of the authors to collect good information in the manuscript from the unique part of the Nepal Himalayan region. I also accept the study to be considered in the journal for publication. However, I have some comments to the authors to be addressed if possible, for quality of MS and its wide readership.

Comments

2.2 Survey and data collection

• In semi-structured questionnaire interviews, what was possible questions asked from the villages to be added in methodology.

The subjects for questionnaire interview were elucidated in the revised MS. In addition, questionnaire was provided as supplementary file for greater details. Questionnaire was provided as supplementary file (S1 Table).

• In the interview, the participants were asked to list the three most important plants for traditional healing. What was the aim behind it?

In order to avoid the data redundancies and to maintain the data consistency, we only asked three most important ethnomedicinal plants in each respondent. This could help sort out the highly valued medicinal plants in traditional culture. 

• Voucher number of each identified plant species to be given in the manuscript.

Thank you for suggestion. We have now provided respective voucher code(s) of each species used in this study. 

2.3 Data analysis

• Matching information (use reports) from at least three respondents was considered a common response for quantitative analysis. I hope use report is related to informants responses for plants as medicine. How it is related to quantitative analysis?.

In the interview, the participants were asked to list the three most important medicinal plants and their use details for traditional healing. From 58 ethnomedicinal plant species mentioned by 76 participants, a total of 419 emic use reports were reported. The use reports were grouped into 55 use types and 14 use categories for convenient analysis. Each species was reported for 2–43 use reports and each respondent recorded 1-12 use.

• Emic use types and etic categories to be described,

Described using Martin (1995). 

• Ecologically salient species to be described.

Described as suggested.

3.3 Agreement on species consensus

• I am not sure how the Quadrant A, B, C, D were assessed.

Second paragraph of section 3.3 described each quadrant. 

3.4 Plant use knowledge and socioeconomic variables

• Limited access to health centres compels people to use local resources for their primary health care. This is appropriate to say that the resources limitation affect its use.

Thank you for suggestion. We paraphrased as suggested. 

• The medicinal plant collection was also influenced by food availability, household economy.

• If authors suggesting insufficient food to the villagers available. What is the reason for insufficient food? and what actually they are collecting to fulfill their needs from the nearby forests to be explained for provide more information of the study area.

Third paragraph of the section 3.4 describes. There is little arable land and it is curtailed by the hills and steep terrain.

3.5 Plant salient features and use values

• There was weak relationship with plant use values and plant salient features. In what way the weak relationship to be explained.

Weak association of plant use values and salience index may be outweighed by the direct harvesting and/or by the popularity of the particular uses of that species.

Conclusion

• Conclusion section to be improved.

Revised and improved as suggested.

Attachment Submitted filename: Response to reviewers.12.18.docx Click here for additional data file.

# Editor report: Decision Letter 1

**DOI:** 10.1371/journal.pone.0246390.r003

**Authors:** Rainer W. Bussmann

18 Jan 2021

Factors that influence the plant use knowledge in the middle mountains of Nepal

PONE-D-20-31088R1

Dear Dr. Kunwar,

We’re pleased to inform you that your manuscript has been judged scientifically suitable for publication and will be formally accepted for publication once it meets all outstanding technical requirements.

Within one week, you’ll receive an e-mail detailing the required amendments. When these have been addressed, you’ll receive a formal acceptance letter and your manuscript will be scheduled for publication.

An invoice for payment will follow shortly after the formal acceptance. To ensure an efficient process, please log into Editorial Manager at http://www.editorialmanager.com/pone/, click the 'Update My Information' link at the top of the page, and double check that your user information is up-to-date. If you have any billing related questions, please contact our Author Billing department directly at authorbilling@plos.org.

If your institution or institutions have a press office, please notify them about your upcoming paper to help maximize its impact. If they’ll be preparing press materials, please inform our press team as soon as possible -- no later than 48 hours after receiving the formal acceptance. Your manuscript will remain under strict press embargo until 2 pm Eastern Time on the date of publication. For more information, please contact onepress@plos.org.

Kind regards,

Rainer W. Bussmann

Academic Editor

PLOS ONE

Additional Editor Comments (optional):

Reviewers' comments:

# Editor report: Acceptance letter

**DOI:** 10.1371/journal.pone.0246390.r004

**Authors:** Rainer W. Bussmann

25 Jan 2021

PONE-D-20-31088R1 

Factors that influence the plant use knowledge in the middle mountains of Nepal 

Dear Dr. Kunwar:

I'm pleased to inform you that your manuscript has been deemed suitable for publication in PLOS ONE. Congratulations! Your manuscript is now with our production department. 

If your institution or institutions have a press office, please let them know about your upcoming paper now to help maximize its impact. If they'll be preparing press materials, please inform our press team within the next 48 hours. Your manuscript will remain under strict press embargo until 2 pm Eastern Time on the date of publication. For more information please contact onepress@plos.org.

If we can help with anything else, please email us at plosone@plos.org. 

Thank you for submitting your work to PLOS ONE and supporting open access. 

Kind regards, 

PLOS ONE Editorial Office Staff

on behalf of

Dr. Rainer W. Bussmann 

Academic Editor

PLOS ONE
